# Hyperpolarized ^13^C-bicarbonate production correlates with excitatory neuron distribution in the human brain

**DOI:** 10.1162/IMAG.a.1342

**Published:** 2026-09-01

**Authors:** Biranavan Uthayakumar, Dylan A. Dingwell, Nicole I.C. Cappelletto, Nadia D. Bragagnolo, Albert P. Chen, Nathan Ma, Ruby Endre, Jesse Gillis, Hany Soliman, Charles H. Cunningham

**Affiliations:** Department of Medical Biophysics, University of Toronto, Toronto, ON, Canada; Physical Sciences, Sunnybrook Research Institute, Toronto, ON, Canada; GE Healthcare, Toronto, ON, Canada; Pharmacy, Sunnybrook Health Sciences Centre, Toronto, ON, Canada; Department of Physiology, University of Toronto, Toronto, ON, Canada; Radiation Oncology, Sunnybrook Health Sciences Centre, Toronto, ON, Canada

**Keywords:** human brain, hyperpolarized ^13^C MRI, cortical metabolism, cell-type distribution, spatial transcriptomics

## Abstract

Whole-brain metabolic topography measured with hyperpolarized ^13^C-pyruvate MRI in 40 healthy individuals was correlated with microarray transcriptomic data from the Allen Human Brain Atlas. Spatial autocorrelation was addressed using both model-based and non-model-based methods, and two parcellation atlases with different numbers of regions were employed to reduce the likelihood of false positives. Brain regions with higher expression of transcripts characteristic of excitatory neurons showed an elevated bicarbonate-to-pyruvate ratio, indicating a higher flux through pyruvate dehydrogenase. Specifically, the Ex2 and Ex4 excitatory neuron subtypes showed the strongest enrichment among all cell-type gene sets. Collectively, these findings connect regional variations in metabolic neuroimaging profiles to underlying patterns of cellular gene expression, offering a framework for understanding the molecular basis of tissue-level metabolic organization.

## 1 Introduction

Glucose metabolism is the brain’s primary energy source, fueling key processes such as neurotransmission, maintenance of ion gradients, and glial and neuronal function (Dienel, 2019). The predominant metabolic pathway in cerebral energy metabolism is the breakdown of glucose into pyruvate, which then enters the TCA cycle and is oxidized via oxidative phosphorylation. However, different brain cell types exhibit distinct metabolic profiles. For example, neurons primarily rely on glucose uptake to sustain their high and constant energy demands (Dienel, 2019; Magistretti & Allaman, 2015), but glutamatergic neurons receive additional support from astrocyte-derived lactate during periods of elevated activity (Magistretti & Pellerin, 1999). The distribution of cell types is heterogeneous throughout the brain, with different regions having different proportions of various types of glial and neuronal cells (Lake et al., 2016, 2018). Consequently, regional patterns of brain metabolism, in part, reflect the aggregated metabolic profiles of cellular subpopulations (Camandola & Mattson, 2017; Kleinridders et al., 2018).

HP-^13^C MRI is a novel metabolic imaging technique that enables imaging of an administered ^13^C-labeled metabolite and downstream products. As the ^13^C-labeled metabolite is converted into downstream metabolites, the ^13^C signal is preserved and can be imaged. In the case of ^13^C-pyruvate, it can be converted into ^13^C-lactate or into acetyl-CoA, producing ^13^C-bicarbonate. HP-^13^C MRI and ^13^C-pyruvate have been used to image numerous organ systems (reviewed in Kurhanewicz et al. (2019)), including the human brain of both healthy volunteers and patients (Lee et al., 2021, 2020; Miloushev et al., 2018; Park et al., 2018). Preliminary results from preclinical studies in disease models indicate that HP-^13^C MRI may be a promising tool to study disorders of the brain (Hackett et al., 2023) and neuroinflammation (Guglielmetti et al., 2023, 2017). Notably, the ^13^C-metabolite distribution in the brain is remarkably consistent across different individuals (Lee et al., 2020). Analysis of these HP-^13^C MRI data using transcriptomic resources, such as the Allen Human Brain Atlas (AHBA), provides an opportunity to interpret regional metabolic patterns in terms of underlying cellular and molecular organization.

The AHBA is a dataset of microarray transcriptomic data collected from six healthy volunteers, designed to characterize the transcriptomic architecture of the human brain (Hawrylycz et al., 2012). The AHBA dataset includes spatial coordinates for each sampled tissue, enabling spatial localization of transcript levels. These transcriptomic data can then be aggregated for gene set enrichment analysis (GSEA) (Subramanian et al., 2005) to derive cell-type information from imaging data (Lake et al., 2016, 2018).

In this study, HP-^13^C MRI data from 40 healthy volunteers were correlated with regional transcriptomic data from the AHBA atlas to identify a transcriptionally defined cellular architecture associated with metabolic topography.

## 2 Materials and Methods

### 2.1 Participants

Written informed consent was obtained from 40 healthy volunteers (17 male, 23 female, mean age 38, median age 28). All procedures were performed under a protocol approved by the Sunnybrook Research Institute Research Ethics Board and by Health Canada. All volunteers were screened for cognitive impairment using the Montreal Cognitive Assessment (Nasreddine et al., 2005). Demographic-level analysis (e.g., sex) was not conducted due to concerns about subsampling in a limited sample.

### 2.1.1 ^13^C-pyruvate preparation and injection

^13^C-pyruvic acid polarization and ^13^C-metabolic imaging was done using previously described workflows (Lee et al., 2020; Uthayakumar et al., 2023). 0.1 mmol/kg of [1-^13^C]-pyruvate were prepared within a sterile fluid path by the pharmacist. Doses consisted of 1.47 g of [1-^13^C]-pyruvic acid (Sigma Aldrich, St. Louis, MO) and the electron paramagnetic agent AH111501 [Tris(8-carboxy-2,2,6,6 (tetra(methoxyethyl) benzo-[1,2-4,5]bis-(1,3)dithiole-4-yl)methyl sodium salt] (Syncom, Groningen, The Netherlands) at a weight ratio of 49:1 wt. The [1-^13^C]-pyruvic acid solution was polarized in a GE SPINLab polarizer system for a minimum of 3 hours to maximize polarization. Immediately before ^13^C-imaging the [1-^13^C]-pyruvic acid sample was dissolved with 38 mL of pressurized, hot, sterile water and transferred to a receiver vessel containing 17.5 mL of neutralizing solution (600 mmol/L NaOH, 333 mmol/L Tris base, and 333 mg/L disodium EDTA) and 19 mL of sterile water through a 65 mm sterilizing filter. A small portion of the resulting ^13^C-pyruvate was used for quality control testing of pH, temperature, polarization level, and AH111501 concentration. Once the solution passed quality control checks, the remainder was transferred into a Medrad (Medrad, Indianola, PA) syringe and injected through a vent filter at 4 mL/s using a Specetris Solaris power injector. This was followed by a 25 mL saline flush at 5 mL/s. ^13^C-image acquisition was initiated at the end of the saline flush.

#### 2.1.2 Image acquisition and reconstruction

Anatomical T1-weighted images (axial fast spoiled gradient echo) with FOV 25.6 × 25.6 cm^2^, TR/TE 7.6 ms, and flip angle of 11° were acquired for each participant to allow for brain-wise localization of ^13^C-image signals during statistical analysis. Anatomical images were acquired with a dedicated 8-channel ^1^H neurovascular array (Invivo Inc., Pewauke, WI).

Whole-brain ^13^C-lactate, ^13^C-bicarbonate, and ^13^C-pyruvate images were acquired using a 3T MRI scanner and a dedicated ^13^C-head coil, built in-house. During the swapping of head coils between ^1^H and ^13^C acquisitions, care was taken to ensure the participant’s head remained stationary. This included using padding along the headrest to minimize movement. Transmit gain calibration was done using a gadolinium-doped urea phantom, and ^13^C-lactate center frequency was calculated relative to the center frequency of ^1^H reported by the scanner. Calibrated transmit gain and center frequency values were used as inputs for the spectrally-selective echo-planar sequence used for ^13^C-image acquisition (Cunningham et al., 2008; Geraghty et al., 2018). The ^13^C-image sequence switches between the center frequencies of ^13^C-lactate, ^13^C-bicarbonate, and ^13^C-pyruvate every 1.2 seconds, acquiring the whole-brain volume at each time point using 24 excitations with net tip angles of 77° for ^13^C-lactate and ^13^C-bicarbonate, and 11° for ^13^C-pyruvate. This process repeats 12 times at 5-second intervals within a 60-second acquisition window. ^13^C-metabolite image FOV was 128 × 16 × 24 voxels with a 1.5 cm isotropic spatial resolution for each metabolite.

Before acquisition of the ^13^C-metabolite images, with all head coils removed, a reference scan using the MRI body coil was acquired at the ^1^H frequency. This reference scan was used to correct for B_0_ inhomogeneities and geometric distortions, thereby improving spatial alignment during reconstruction in MATLAB (Geraghty et al., 2018; MATLAB, 2019). Distortion-corrected ^13^C-metabolite images were then resampled to the resolution and FOV of the anatomical T1-weighted images and summed across time points to increase SNR. The final ^13^C-lactate, ^13^C-bicarbonate, and ^13^C-pyruvate images were used for all subsequent analysis.

### 2.2 Statistical analysis

#### 2.2.1 HP-^13^C image analysis

Anatomical T1 images were registered to MNI152 space using FSL (Andersson et al., 2007; Mazziotta et al., 1995; Woolrich et al., 2009) and the transformation matrix from the resulting MNI152 registration was then applied to the ^13^C-metabolite images. These registered images were then voxel-wise averaged across participants to produce group-average images for ^13^C-lactate, ^13^C-bicarbonate, and ^13^C-pyruvate. Finally, these group-average ^13^C-metabolite images were overlaid with the parcellated Schaefer 200 atlas (100 parcellations per hemisphere) (Schaefer et al., 2018; Yeo et al., 2011) from the Yeo lab (https://github.com/ThomasYeoLab/CBIG) to compile regional average ^13^C-metabolite signals. The following metabolite ratio maps were then computed through region-wise division of each of the corresponding ^13^C-metabolites: ^13^C-lactate/^13^C-bicarbonate ratio (L*_B_*), ^13^C-lactate/^13^C-pyruvate ratio (L*_P_*) and the ^13^C-bicarbonate/^13^C-pyruvate ratio (B*_P_*). Raw, normalized signals were also tested in subsequent analyses (see Supplemental Material section 0.2 and 0.3, Supplementary Tables S3, S4, S7, and S8).

The Connectome Workbench was used to generate surface maps of the B*_P_* ratio, both before and after parcellation with the Schaefer 200 atlas, for visualization purposes (Marcus et al., 2011).

#### 2.2.2 AHBA microarray data processing

Microarray data obtained from the AHBA database (n = 6, ages 24–57) (Hawrylycz et al., 2012; Sunkin et al., 2012) was pre-processed using the Abagen python package (Arnatkeviciūtė et al., 2019; Hawrylycz et al., 2012; Markello et al., 2021) (available from https://doi.org/10.5281/zenodo.3451463). The Abagen package was used to remove low-SNR microarray gene data, aggregate by probe, normalize within donors, and average by region and across donors. This resulted in a filtered transcript expression dataset for 15,633 genes, annotated at the per-parcel level for the Schaefer 200 atlas. Only data from the left hemisphere were used, as the right hemisphere had transcript data from only two donors.

#### 2.2.3 Correlation analysis

**Fig. 1.**
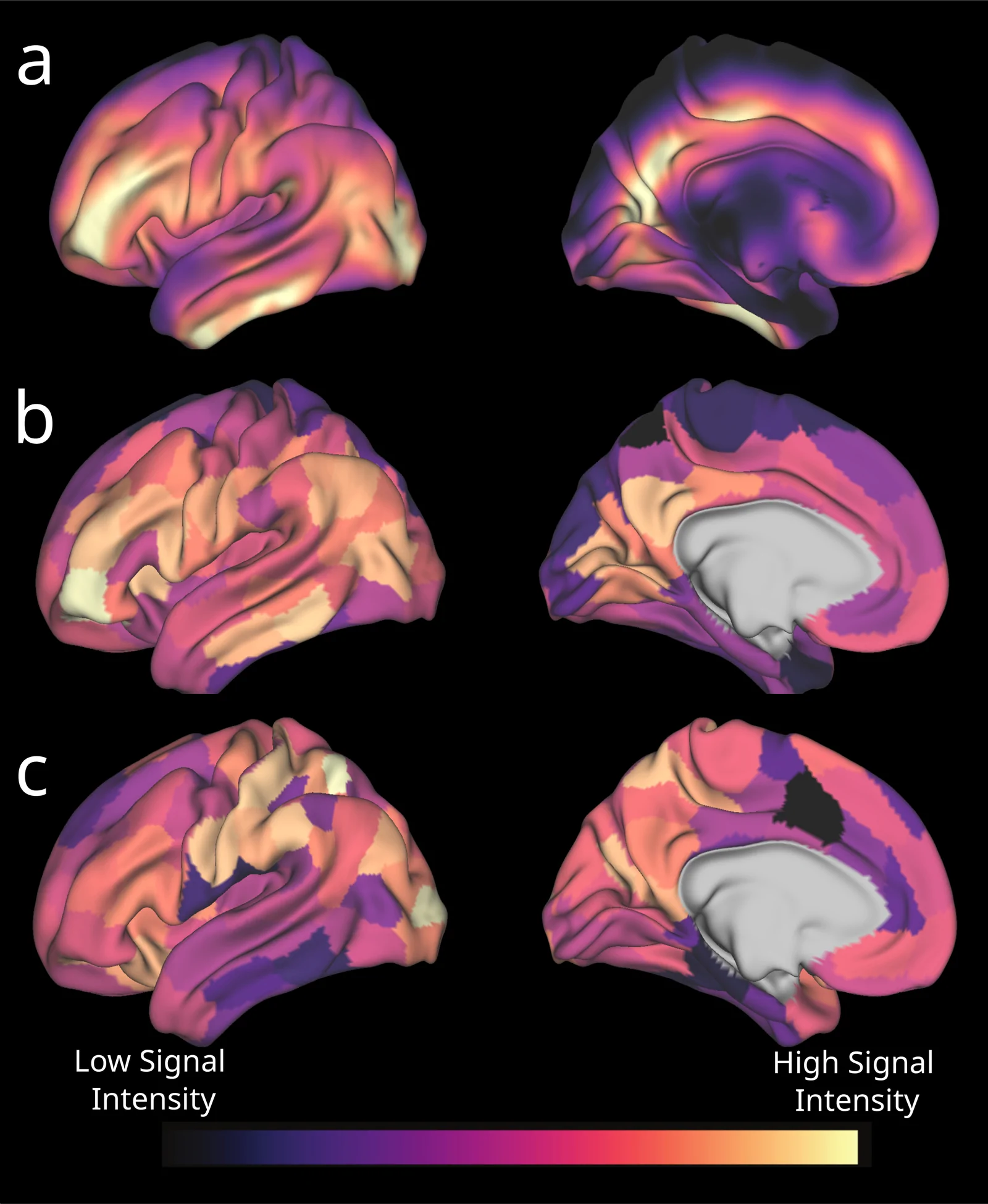
Relative signal intensities for: (a) Unparcellated surface map for the mean B*_P_* ratio of the n = 40 healthy participants scanned. (b) Mean regional B*_P_* ratio for the n = 40 healthy participants parcellated using the Schaefer 200 parcellation atlas. (c) Example spatial autocorrelation preserving surrogate map of the mean B*_P_* ratio.

To test for associations between individual genes and ^13^C-ratios, Spearman correlations were calculated, with p-values computed using permutation testing (Burt et al., 2020; Viladomat et al., 2014). This method accounts for spatial autocorrelation, which describes the correlation between a measurement and itself as a function of spatial distance, and can lead to erroneously significant correlations if similar spatial autocorrelation structures are present in two different spatial maps (Alexander-Bloch et al., 2018; Burt et al., 2018; Fulcher et al., 2021). The effect of spatial autocorrelation can be controlled for by generating permutations of the averaged empirical ^13^C-ratio maps (Burt et al., 2018). This avoids relying on distributional assumptions, since significance is derived directly from the data. Example model-based maps, created using the variogram method (Burt et al., 2020), are shown in Figure 1. Permutation maps for the Schaefer 200 parcellations were generated for each ^13^C-ratio map and used separately to test correlations for individual genes. In total, 15,633 genes were tested for significant correlations with each of the ^13^C-ratio maps, and the statistical tests were two-sided. In brief, model-based null distributions were generated using the brainSMASH algorithm, which quantifies spatial autocorrelation in each ^13^C-ratio map via a variogram. Mean brain region ^13^C-ratio map values were then shuffled randomly. These mean brain-region ^13^C-ratio values were then blurred to produce a new variogram that closely matched the original variogram through iterative optimization. The brainSMASH package was used to create 1,000 permutations of each ^13^C-ratio brain map. Correlations were calculated for each permutation with each gene, producing a null distribution for each gene and each ratio brain map. Significance was calculated as the percentage of correlations in the corresponding null distribution that exceeded the empirical Spearman correlation. Correlation *p*-values were thresholded based on a false-discovery rate (FDR) threshold of 0.05. The same workflow was applied to the Schaefer 100 atlas (50 regions per hemisphere) as well to compare the effect of parcel count on correlation results (see Supplemental Material section 0.3, Supplementary Tables S5 and S6).

### 2.3 Gene set enrichment analysis

**Fig. 2.**
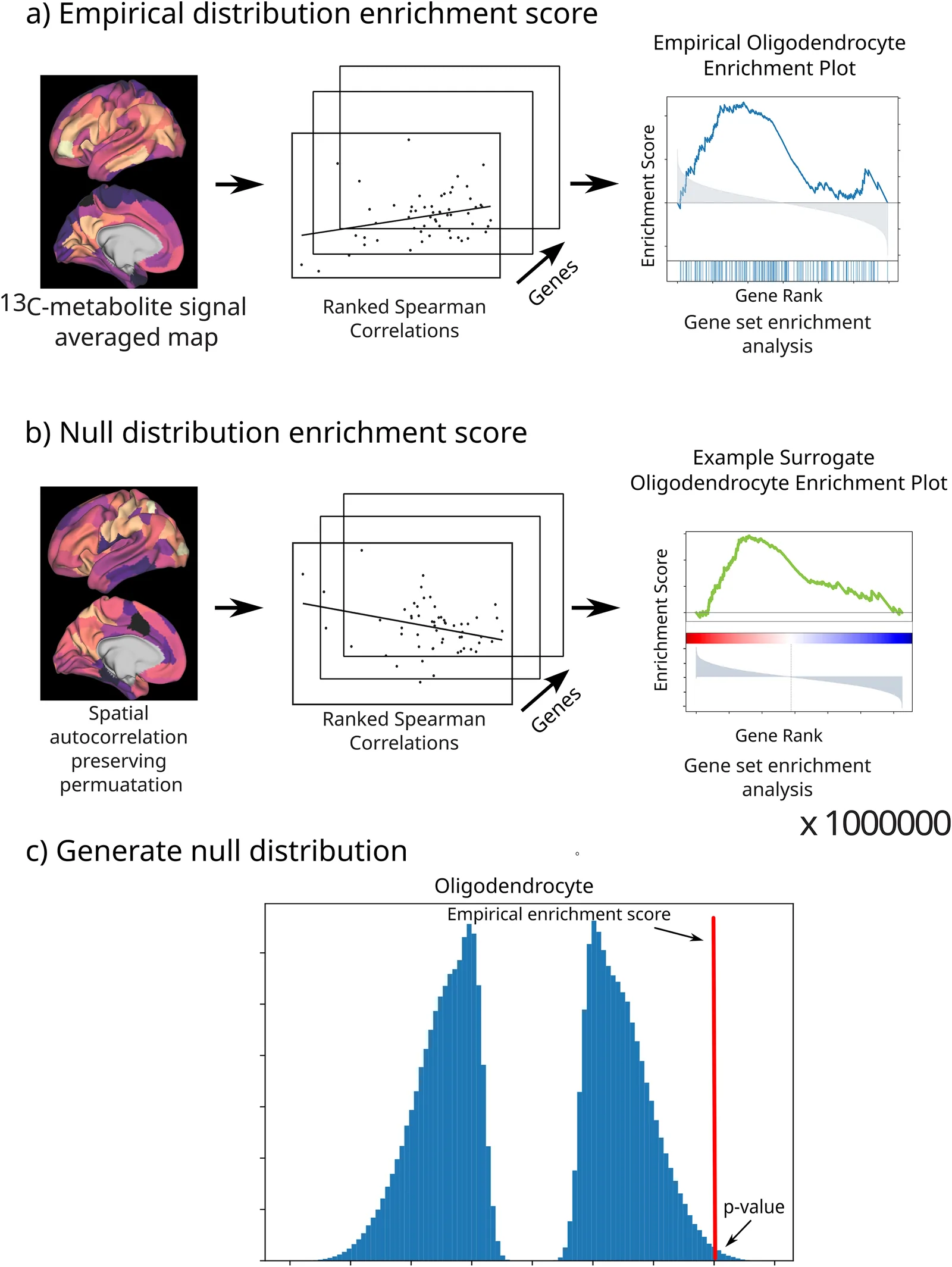
Workflow for calculating the empirical enrichment score and the null distribution of enrichment scores. (a) Empirical enrichment scores use the Spearman correlation between each gene and an across-person averaged ^13^C-ratio map. (b) Null distribution enrichment scores are calculated by using a spatial autocorrelation-preserving permutation of the across-person averaged ^13^C-ratio map. (c) Final *p*-values are calculated by comparing the empirical enrichment score to the null distribution of enrichment scores.

A limitation of mass univariate correlation is the difficulty of aggregating results to draw unbiased conclusions. Gene set enrichment analysis (GSEA) helps address this problem by grouping correlations into functional groups (Subramanian et al., 2005). We used cell-type gene sets (Lake et al., 2016, 2018) to aggregate gene-wise correlations into 30 cell types. GSEA calculates enrichment scores (ES) by moving down a list of genes ranked by Spearman correlation (from 1 to -1). Whenever a gene belongs to the set being tested, the enrichment score (ES) is incremented; otherwise, it is decremented. The final ES is the maximum absolute deviation from zero and was calculated using the prerank function of the GSEApy package (Fang et al., 2023). Gene sets characteristic of 13 different excitatory neurons, 11 different inhibitory neurons, oligodendrocytes, astrocytes, endothelial cells, microglia, oligodendrocyte progenitor cells, and pericytes were tested (Lake et al., 2018). To compute *p*-values, one million permutations of the ^13^C-ratio maps were created with the brainSMASH package, and each was subjected to the full GSEA analysis to compute a null distribution of ES. One million permutations were selected to produce consistent results across repeated runs of the analysis. This stability in results was not observed with a permutation count of 100,000, where repeat runs led to enrichment in different cell types. An enrichment score (ES) was calculated for each permutation and used to build a null distribution for comparison to the actual enrichment scores. Note that, in contrast to the calculation of the significant Spearman correlations, spatial autocorrelation was accounted for at the level of cell type and not at the level of gene, as each permutation was used to calculate an enrichment score. The final cell-type *p*-values were thresholded at an FDR threshold of 0.25 (Benjamini & Hochberg, 1995). A summary diagram of the methodology is shown in Figure 2.

To test whether the specific null distribution generation technique affects the GSEA results, a spin permutation null distribution generation method was also used: the spatial-permutation spin test (Váša et al., 2018). Parcel centroid coordinates were calculated using the NetNeuroTools package (Markello et al., 2022) and 100,000 spatial-autocorrelation preserving permutations were generated. Each of these permutations was then used to calculate enrichment scores for the 30 brain cell types, building the null distribution for each cell type. See the Supplemental Material Tables S1 and S2 under section 0.1 for a full list of model-based and spin permutation-based ^13^C-ratio GSEA results. The model-based and spin test null distribution generation techniques were also used with the Schaefer 100 atlas to test for the effect of parcel count on GSEA results (see Supplemental Material section 0.3).

## 3 Results

### 3.1 Correlations between metabolic topography and gene expression

Example maps for the Schaefer 200 atlas are shown in Figure 1. Gene-level correlations between each gene and ^13^C-ratio maps were computed, and spatial autocorrelation was accounted for using model-based null distribution generation. Correlation strengths were consistent between Schaefer 200 and Schaefer 100 atlases.

**Fig. 3.**
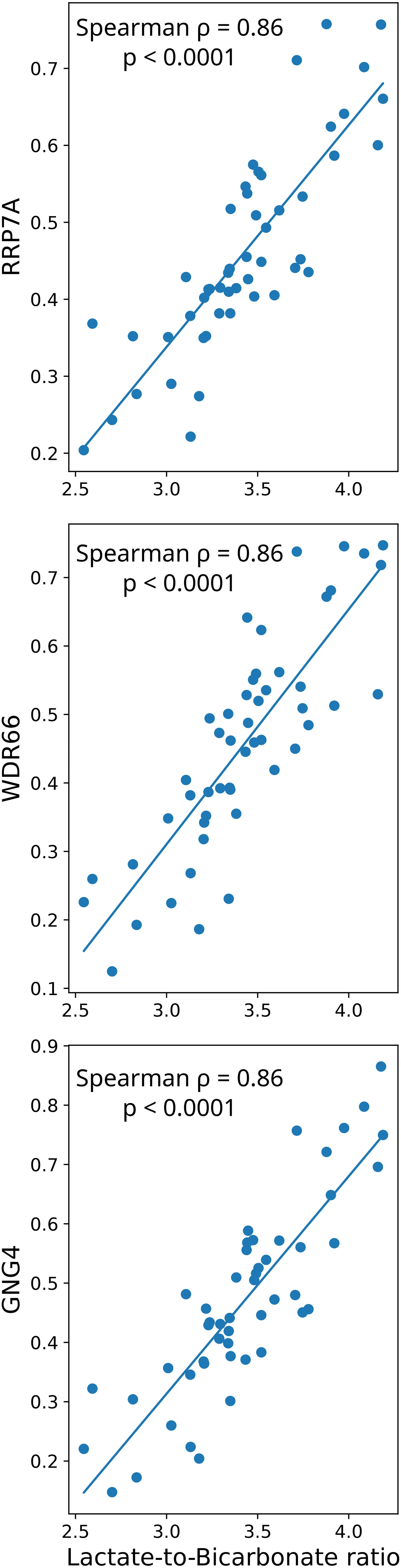
The three most positive correlations between gene transcript expression and the L*_B_* ratio brain map. The results shown are for the Schaefer 200 atlas.

**Fig. 4.**
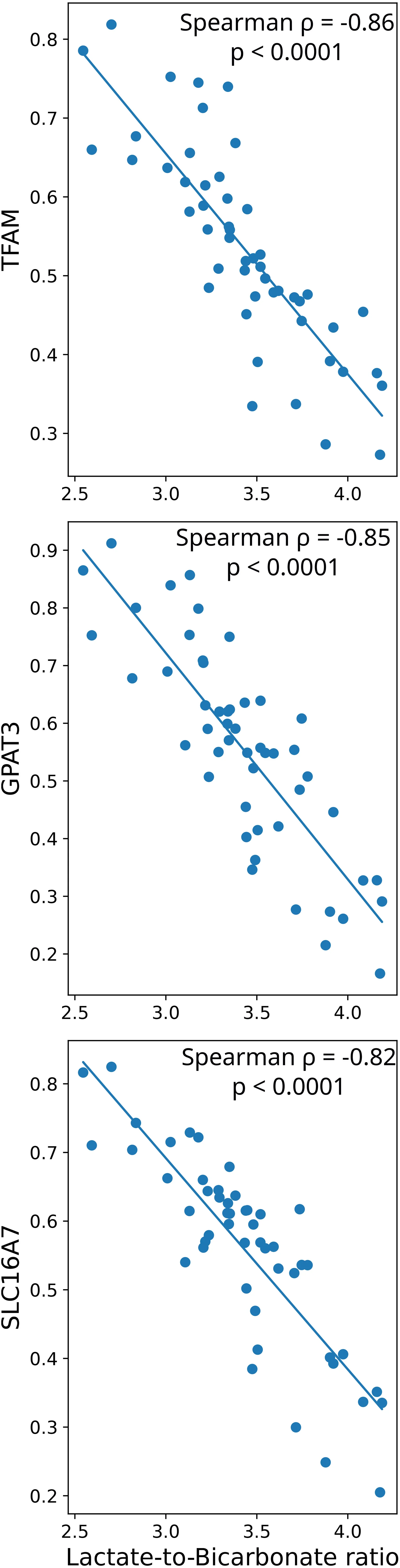
The two most negative correlations between gene transcript expression and the L*_B_* ratio brain map. A gene of interest, SLC16A7, which encodes the MCT2 transporter, is also highlighted. The results shown are for the Schaefer 200 atlas.

The three genes most positively and most negatively correlated with the L*_B_* ratio, which had the strongest correlations among all ratios, are plotted in Figures 3 and 4, respectively.

Monocarboxylate transporters (MCT1, MCT2) are the rate-limiting step for the transport of ^13^C-pyruvate and production of ^13^C-lactate in cancer cells (Granlund et al., 2020). Based on this, it was hypothesized that MCT distribution would correlate with the distribution of ^13^C-ratios. SLC16A7, the gene encoding the neuron-specific MCT2 was significantly correlated with the L*_B_* ratio as seen in Figure 4 (Spearman ρ = -0.72 *p* = 0.003). The strong negative correlation between the L*_B_* ratio and MCT2 transporter expression suggests that regions with higher ^13^C-bicarbonate production relative to ^13^C-lactate production tend to have higher transporter expression. No other ^13^C-ratios were found to be significantly correlated with any of the MCT transporters.

### 3.2 Gene set enrichment analysis of B*_P_* ratio maps reveals neuronal specificity

**Fig. 5.**
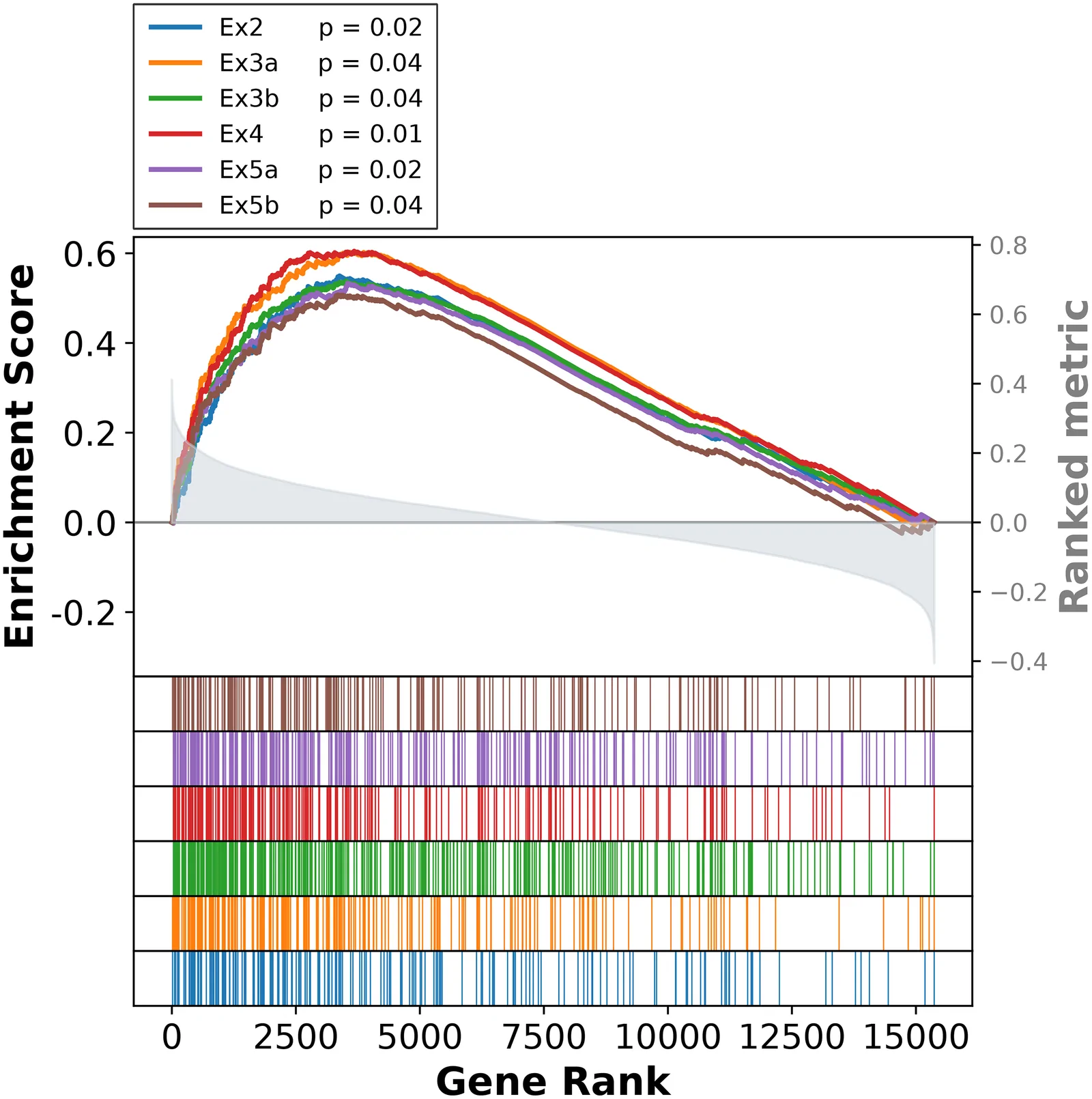
Enrichment plot for the B*_P_* ratio map showing statistically significant enrichment for the Ex2, Ex3a, Ex4, Ex5a, and Ex5b excitatory neuron cell types using the Schaefer 200 parcellation. All results survive FDR-thresholding.

Of the three ^13^C-ratios tested only the B*_P_* ratio showed significant enrichment when tested with two-sided model-based spatial autocorrelation-preserving null models, with positive enrichment for the Ex2 (p = 0.02), Ex3a (p = 0.04), Ex3b (p = 0.04), Ex4 (p = 0.01), Ex5a (p = 0.02), and Ex5b (p = 0.04) excitatory neurons. An example enrichment plot for the Schaefer 200 atlas is shown in Figure 5, and a summary table of results with model-based permutation generation is provided in Table 1. Results surviving FDR-thresholding at a rate of 0.25 are in bold.

**Table 1.** B*_P_* ratio cell enrichments and associated significance values for a model-based null distribution with the Schaefer 200 atlas.

Cell Type	Enrichment score	p-value
Ex2	0.55	**0.015**
Ex3a	0.60	**0.035**
Ex3b	0.54	**0.043**
Ex4	0.60	**0.014**
Ex5a	0.53	**0.022**
Ex5b	0.51	**0.039**

Significant p-values surviving FDR-thresholding at a rate of 0.25 are listed.

As a comparison, *p*-values were also computed using a null distribution generated using a two-sided spin permutation method (Váša et al., 2018). Enrichment statistical significance for cell types that were significant with the spin test-based technique is listed in Table 2.

**Table 2.** B*_P_* ratio cell enrichments and associated significance values for the spin permutation-based null distribution with the Schaefer 200 atlas.

Cell Type	Enrichment score	p-value
Ex2	0.55	**0.025**
Ex3a	0.60	**0.050**
Ex3b	0.54	**0.095**
Ex4	0.60	**0.15**
Ex5a	0.53	**0.077**
Ex5b	0.51	**0.051**
Ex6a	0.41	**0.092**

Significant p-values surviving FDR-thresholding at a rate of 0.25 are listed.

## 4 Discussion

In this study, the correlation between cell-type distribution and group-averaged metabolic topography was assessed using HP-^13^C MRI. To account for the numerous sources that can imprint spatial structure and lead to spatial dependence between nearby regions, including partial-voluming and coil sensitivity patterns, spatial autocorrelation correction techniques were used. The topographic pattern of B*_P_*, a marker of flux from pyruvate to acetyl-CoA (relative to regional ^13^C-pyruvate uptake), was enriched most strongly for specific excitatory neuron subtypes. The remaining excitatory neurons, 11 inhibitory neurons, and six glial cells did not show significant enrichment. Although these results did not always survive FDR correction, a consistent pattern of findings was observed across both parcel sets and across model-based and spin permutation-based null models. Specifically, excitatory neuron gene sets were reliably enriched in the whole-brain B*_P_* ratio map, whereas no enrichment was consistently detected for any other ^13^C-metabolite maps.

Spearman correlations between each of the 15,633 genes and the regional ^13^C-lactate, ^13^C-bicarbonate, ^13^C-pyruvate, as well as L*_P_*, B*_P_*, and L*_B_* ratios were calculated. Only L*_P_* and L*_B_* exhibited strong Spearman correlations with individual transcripts, although neither showed consistent enrichment for any cell type. A number of these correlations were strong and statistically significant, notably the correlation between the L*_B_* ratio and the gene encoding the MCT2 transporter, which is primarily expressed in neurons and mediates monocarboxylate uptake. However, no correlation was found between the LDHA or LDHB enzymes and any of the ^13^C-ratios, which catalyze the forward and backward conversion of pyruvate into lactate, respectively. Given MCT2’s high affinity for lactate, it may transport lactate into cells that would not otherwise produce significant amounts of lactate. While this hypothesis is worth testing, it also highlights that interpreting results from single-gene-level correlations is not straightforward.

Ex2, Ex3, Ex4 and Ex5 excitatory neurons were consistently enriched under both the model-based and spin permutation-based null distribution generation methods for both the Schaefer 100 and 200 atlases. Ex2, Ex3, and Ex4 neurons are all layer 4 neurons found throughout the frontal and visual cortices (Lake et al., 2018), while Ex5 neurons are layer 5 neurons with high metabolic demand stemming from signalling and maintaining ion gradients, like Ca^2^ + gradients, along the expansive dendritic networks that these neurons possess (Hyder et al., 2013; Yi et al., 2019). The signaling energy requirements of Ex5 neurons, in particular, are likely to persist in the awake resting-state (Hyder et al., 2013; Yu et al., 2018) and help explain the consistently large output of ^13^C-bicarbonate signal that has been observed in prior studies during this state (Lee et al., 2020). Layer 5 neurons are found throughout the cortex, particularly in transmodal regions such as the limbic network, with their density decreasing toward the visual cortex (Zhang et al., 2025). All of the consistently enriched neurons are intratelencephalic, meaning they project their axons within the telencephalon, which encompasses the cortex, striatum and related structures (Reiner et al., 2010). Layer 4 intratelencephalic neurons receive thalamocortical signals, such as sensory or motor inputs, and project them to layers 2 and 3, where they are transported to layers 5 and 6 to generate output signals. Cytochrome oxidase staining, a marker of stable oxidative metabolism (Wong-Riley, 1989), is highest in layer 4 neurons (Anderson et al., 2009; Kageyama & Wong-Riley, 1986), in agreement with our findings of the greatest B*_P_* ratio in regions with large populations of layer 4 and layer 5 neurons.

Somewhat surprisingly, the strongest positive correlation between any gene transcript and the B*_P_* ratio is 0.41, indicating that individual gene correlation strength is insufficient to account for regional variance. Instead, it is the pronounced alignment of hundreds of genes, which is positively correlated with the B*_P_* ratio across all tested genes, that indicates that this cell type consistently contributes to spatial variation. In contrast, the L*_P_* and L*_B_* ratios both exhibited several genes with strong individual correlations. However, the aggregate of these correlations did not align with any particular cell type, as enrichment requires synergistic correlation patterns rather than solely correlation strength.

Since the L*_B_* ratio is a readout of the ^13^C-lactate production relative to oxidative metabolism of ^13^C-pyruvate, it might be expected that similar enrichment results would be seen for this ratio as well. However, the L*_B_* ratio correlates poorly with B*_P_* distribution patterns (Blazey et al., 2025). In that same study (which used most of the same HP-^13^C neuroimaging data as this study), L*_P_* correlates quite strongly with B*_P_* with a correlation coefficient of 0.86. The results presented in this paper thus seem hard to reconcile with the high correlation between the B*_P_* and L*_P_* ratios, since L*_P_* would be expected to show enrichment in a similar set of cell types as B*_P_* given this high correlation. However, the lack of a perfect correlation means that patterns in the B*_P_* ratio can reinforce themselves across hundreds of gene correlations, resulting in enrichment that is not present in the L*_P_* ratio. This is a key strength of GSEA: as long as each ^13^C-metabolite and ratio map provides unique information, GSEA can highlight subtle patterns by leveraging the collective signal across large gene expression datasets. Finally, while the B*_P_* distribution pattern aligns most closely with those of excitatory neurons, it cannot be concluded that these excitatory neurons are the dominant site of this oxidative consumption of pyruvate. Rather, it can be concluded that in regions with high excitatory neuron density, the highest production rates of ^13^C-bicarbonate relative to ^13^C-pyruvate uptake are observed.

Since this analysis was done at the regional level, B*_P_* should be considered an average measure of intracellular ^13^C-bicarbonate production relative to ^13^C-pyruvate uptake, influenced by several factors. ^13^C-pyruvate uptake is likely rate-limited by vascular monocarboxylate transporter expression (Granlund et al., 2020), implying that cerebral blood volume and vascular reactivity contribute to regional variability. ^13^C-bicarbonate, on the other hand, primarily reflects intracellular pyruvate dehydrogenase activity, modulated by monocarboxylate transporter expression levels. The coarse resolution of HP-^13^C MRI means that certain regions will approach the size of a single voxel, introducing signal contamination across adjacent regions. Nevertheless, while spatial autocorrelation correction and the use of averaged ^13^C-metabolite maps minimize this source of variance without weakening the conclusions, it remains an important limitation to keep in mind when assessing individual regions.

In conclusion, this work shows that the HP-^13^C MRI-derived B*_P_* ratio tends to be highest in regions of the brain with high levels of specific excitatory neuron-specific transcripts, suggesting a spatially organized metabolic phenotype of the human brain that aligns with transcriptionally defined cellular architecture.

## Supplementary Material

Supplementary Material

## Data Availability

All correlation and GSEA data, as well as the code to reproduce the figures and results, are available at https://doi.org/10.5281/zenodo.20575695.
