# Supplementary material for: Hyperpolarized 13C-bicarbonate production correlates with excitatory neuron distribution in the human brain
Source: Imaging Neurosci (Camb). 2026 Sep 1;4:IMAG.a.1342. doi: 10.1162/IMAG.a.1342 (PMC13537045; doi:10.1162/IMAG.a.1342)
Supplement: Supplementary file 1 [file IMAG.a.1342_supp.pdf]

Supplemental Material

0.1 Schaefer 200 complete model-based and spin permutation-based GSEA results

Table S1 shows the full GSEA results for all 30 cell types tested for each of B_P, L_P, and L_B ratios. Bold p-values correspond to FDR-thresholded at a rate of 0.25, and p-values were calculated using model-based null distributions for each ratio. Table S2 follows a similar format, but uses spin permutation-generated null distributions.

Table S1: GSEA results for Schaefer 200 ^{13}C-ratios using model-based null distributions.

	B_P		L_P		L_B	
**Cell Type**	**ES**	$\boldsymbol{p}$	**ES**	$\boldsymbol{p}$	**ES**	$\boldsymbol{p}$
Ast	−0.49	0.10	0.26	0.66	0.47	0.066
End	0.34	0.26	−0.19	0.83	−0.32	0.32
Ex1	0.52	0.064	0.27	0.71	−0.27	0.71
Ex2	0.55	**0.015**	0.31	0.49	−0.24	0.74
Ex3a	0.60	**0.036**	−0.27	0.77	−0.49	0.17
Ex3b	0.54	**0.043**	−0.25	0.79	−0.42	0.18
Ex3c	0.46	0.11	0.20	0.83	−0.32	0.40
Ex3d	0.43	0.25	−0.40	0.34	−0.49	0.088
Ex3e	0.40	0.27	0.34	0.44	0.23	0.79
Ex4	0.60	**0.014**	−0.22	0.87	−0.43	0.17
Ex5a	0.53	**0.022**	0.21	0.88	−0.34	0.29
Ex5b	0.51	**0.039**	0.33	0.40	−0.25	0.73
Ex6a	0.41	0.090	0.27	0.54	−0.23	0.74
Ex6b	0.40	0.21	0.34	0.37	0.26	0.70
Ex8	0.42	0.16	0.40	0.20	0.24	0.78
In1a	0.20	0.83	0.28	0.39	0.30	0.30
In1b	0.29	0.36	0.31	0.28	0.24	0.68
In1c	0.28	0.43	0.28	0.42	0.23	0.73
In2	0.28	0.35	0.21	0.76	−0.20	0.89
In3	0.36	0.18	−0.26	0.58	−0.32	0.31
In4a	0.26	0.57	0.32	0.35	0.32	0.31
In4b	0.31	0.18	0.23	0.66	0.21	0.73
In6a	0.29	0.38	−0.31	0.29	−0.34	0.13
In6b	0.27	0.64	−0.42	0.20	−0.42	0.19
In7	0.29	0.43	−0.20	0.89	−0.23	0.78
In8	0.28	0.36	0.28	0.38	0.28	0.31
Mic	−0.28	0.29	0.22	0.59	0.29	0.19
OPC	−0.35	0.27	0.28	0.54	0.40	0.094
Oli	0.23	0.63	0.39	0.24	0.23	0.50
Per	0.28	0.35	0.27	0.36	0.22	0.63

Table S2: GSEA results for Schaefer 200 ^{13}C-ratios using spin permutation-based null distributions.

	B_P		L_P		L_B	
**Cell Type**	**ES**	***p***	**ES**	***p***	**ES**	***p***
Ast	−0.49	**0.040**	0.26	0.43	0.47	0.22
End	0.34	**0.028**	−0.19	0.30	−0.32	0.079
Ex1	0.52	**0.065**	0.27	0.81	−0.27	0.70
Ex2	0.55	**0.025**	0.31	0.62	−0.24	0.43
Ex3a	0.60	**0.050**	−0.27	0.40	−0.49	0.33
Ex3b	0.54	**0.095**	−0.25	0.58	−0.42	0.51
Ex3c	0.46	**0.11**	0.20	0.51	−0.32	0.56
Ex3d	0.43	**0.12**	−0.40	0.66	−0.49	0.52
Ex3e	0.40	**0.13**	0.34	0.80	0.23	0.80
Ex4	0.60	**0.15**	−0.22	0.33	−0.43	0.28
Ex5a	0.53	**0.077**	0.21	0.72	−0.34	0.58
Ex5b	0.51	**0.052**	0.33	0.68	−0.25	0.55
Ex6a	0.41	**0.092**	0.27	0.54	−0.23	0.44
Ex6b	0.40	**0.10**	0.34	0.70	0.26	0.68
Ex8	0.42	**0.11**	0.40	0.92	0.24	0.81
In1a	0.20	0.22	0.28	0.80	0.30	0.64
In1b	0.29	0.32	0.31	0.76	0.24	0.72
In1c	0.28	**0.15**	0.28	0.58	0.23	0.26
In2	0.28	0.27	0.21	0.62	−0.20	0.36
In3	0.36	**0.16**	−0.26	0.30	−0.32	0.41
In4a	0.26	**0.13**	0.32	0.66	0.32	0.56
In4b	0.31	0.28	0.23	0.70	0.21	0.54
In6a	0.29	0.23	−0.31	0.47	−0.34	0.50
In6b	0.27	0.20	−0.42	0.72	−0.42	0.29
In7	0.29	0.30	−0.20	0.46	−0.23	0.21
In8	0.28	0.21	0.28	0.61	0.28	0.69
Mic	−0.28	**0.039**	0.22	0.22	0.29	0.13
OPC	−0.35	**0.15**	0.28	0.40	0.40	0.28
Oli	0.23	**0.12**	0.39	0.57	0.23	0.38
Per	0.28	**0.066**	0.27	0.11	0.22	0.10

0.2 ^{13}C-metabolite brainstem normalization, gene-wise correlations, and model-based GSEA with Schaefer 200 atlas

To assess gene-wise correlations and GSEA using raw metabolites, the first step was to normalize each metabolite signal to a standard region, reducing nuisance between-person variance (e.g., from sample polarization) in the raw values. The brainstem was selected as this reference region, a common practice in FDG-PET imaging, given its consistently uniform metabolic activity across individuals (Verger et al., 2021). To get brainstem values, anatomical T1 images of each participant were processed through the SLANT parcellation algorithm to measure the mean brainstem signal for ^{13}C-lactate, ^{13}C-bicarbonate, and ^{13}C-pyruvate images (Huo et al., 2019). Note that brainstem-normalized ^{13}C-metabolite maps were not used to produce the ratio maps to avoid redundant normalization. Regions as delineated by the Schaefer 200 atlas were then each divided by the corresponding participant's brainstem value to get brainstem-normalized ^{13}C-metabolite maps. Each of these maps for each of the three ^{13}C-metabolites was averaged across the forty subjects to produce mean ^{13}C-metabolite maps. Correlations with model-based spatial autocorrelation correc-

tion and GSEA with model-based and spin permutation-based autocorrelation correction were then run using these ^{13}C-metabolite maps in a fashion identical to the ^{13}C-ratio maps. No significant enrichments were found between any of the individual ^{13}C-metabolite maps and the cell type distribution used in this study. A full list of GSEA results for both model-based and spin permutation-based brainstem normalized ^{13}C-metabolite maps can be found in Table S3 and Table S4, respectively.

Table S3: GSEA results for Schaefer 200 brainstem-normalized ^{13}C-metabolites using model-based null distributions.

	^{13}C-BICARBONATE		^{13}C-LACTATE		^{13}C-PYRUVATE	
**Cell Type**	**ES**	***p***	**ES**	***p***	**ES**	***p***
Ast	−0.46	0.079	−0.37	0.32	−0.31	0.47
End	0.33	0.32	0.30	0.34	0.20	0.76
Ex1	0.36	0.33	0.29	0.66	0.17	0.99
Ex2	0.34	0.21	0.29	0.62	−0.19	0.94
Ex3a	0.54	0.078	0.42	0.44	0.35	0.63
Ex3b	0.35	0.45	−0.14	1.0	0.16	0.99
Ex3c	0.20	0.88	−0.26	0.64	−0.28	0.51
Ex3d	0.50	0.078	0.36	0.49	0.39	0.45
Ex3e	−0.27	0.64	−0.35	0.40	−0.42	0.22
Ex4	0.43	0.19	0.26	0.79	0.24	0.84
Ex5a	0.31	0.43	−0.12	1.0	−0.19	0.92
Ex5b	0.30	0.42	0.24	0.83	−0.22	0.84
Ex6a	0.30	0.34	0.25	0.67	0.19	0.94
Ex6b	0.29	0.61	0.29	0.51	−0.17	0.99
Ex8	0.20	0.91	0.20	0.88	−0.28	0.58
In1a	−0.16	0.98	0.13	1.0	−0.22	0.77
In1b	0.23	0.79	0.23	0.71	0.17	0.99
In1c	0.28	0.42	0.27	0.45	0.22	0.75
In2	0.29	0.26	0.29	0.31	0.26	0.38
In3	0.38	0.094	0.35	0.20	0.31	0.34
In4a	0.19	0.95	0.21	0.84	−0.18	0.96
In4b	0.22	0.65	0.19	0.92	0.17	0.97
In6a	0.33	0.18	0.23	0.66	0.29	0.39
In6b	0.38	0.33	0.23	0.77	0.36	0.44
In7	0.34	0.16	0.31	0.34	0.28	0.44
In8	0.19	0.90	0.17	0.95	−0.19	0.90
Mic	−0.29	0.20	−0.31	0.20	−0.25	0.38
OPC	−0.32	0.40	−0.17	0.96	−0.21	0.88
Oli	−0.10	1.0	0.26	0.58	−0.13	0.97
Per	−0.19	0.81	−0.12	1.0	−0.15	0.94

Table S4: GSEA results for Schaefer 200 brainstem-normalized ^{13}C-metabolites using spin permutation null distributions.

	^{13}C-bicarbonate		^{13}C-lactate		^{13}C-pyruvate	
**Cell Type**	**ES**	***p***	**ES**	***p***	**ES**	***p***
Ast	−0.46	0.29	−0.37	0.76	−0.31	0.82
End	0.33	0.12	0.30	0.26	0.20	0.48
Ex1	0.36	0.57	0.29	0.94	0.17	0.98
Ex2	0.34	0.50	0.29	0.86	−0.19	0.98
Ex3a	0.54	0.34	0.42	0.92	0.35	0.83
Ex3b	0.35	0.70	−0.14	0.99	0.16	0.99
Ex3c	0.20	0.81	−0.26	0.87	−0.28	0.93
Ex3d	0.50	0.33	0.36	0.96	0.39	0.80
Ex3e	−0.27	0.61	−0.35	0.92	−0.42	0.81
Ex4	0.43	0.50	0.26	0.93	0.24	0.97
Ex5a	0.31	0.65	−0.12	0.99	−0.19	1.0
Ex5b	0.30	0.59	0.24	0.86	−0.22	0.98
Ex6a	0.30	0.50	0.25	0.85	0.19	0.98
Ex6b	0.29	0.39	0.29	0.89	−0.17	0.95
Ex8	0.20	0.78	0.20	0.85	−0.28	0.99
In1a	−0.16	0.93	0.13	0.99	−0.22	0.99
In1b	0.23	0.67	0.23	0.83	0.17	0.95
In1c	0.28	0.40	0.27	0.86	0.22	0.97
In2	0.29	0.49	0.29	0.90	0.26	0.89
In3	0.38	0.43	0.35	0.79	0.31	0.88
In4a	0.19	0.78	0.21	0.81	−0.18	0.98
In4b	0.22	0.68	0.19	0.91	0.17	0.98
In6a	0.33	0.44	0.23	0.79	0.29	0.87
In6b	0.38	0.60	0.23	0.71	0.36	0.87
In7	0.34	0.55	0.31	0.92	0.28	0.88
In8	0.19	0.70	0.17	0.93	−0.19	0.98
Mic	−0.29	0.25	−0.31	0.43	−0.25	0.70
OPC	−0.32	0.38	−0.17	0.94	−0.21	0.94
Oli	−0.10	0.99	0.26	0.85	−0.13	0.99
Per	−0.19	0.50	−0.12	0.95	−0.15	0.76

0.3 ^{13}C-metabolite and ^{13}C-ratio gene-wise correlations and GSEA with the Schaefer 100 atlas

To assess the effect of parcel count, a secondary analysis was conducted using the Schaefer 100 atlas, following the same steps as for the Schaefer 200 atlas. Firstly, model-based null distribution generation and subsequent gene-level Spearman correlation testing showed that Spearman correlation strengths were consistent between the Schaefer 200 and Schaefer 100 atlases.

Model-based permutation GSEA results for the Schaefer100 atlas ^{13}C-ratios are shown in Table S5. The Schaefer 100 atlas showed positive enrichment for the Ex2 ($p = 0.04$), Ex5a ($p = 0.03$), and Ex6a ($p = 0.04$) excitatory neurons. However, these do not survive FDR-thresholding, and the parcel count likely affects exactly which excitatory neurons are enriched. Under the spin permutation technique, the Schaefer 100 results are summarized in Table S6. Results with the Schaefer 100 atlas support the findings of the Schaefer 200 atlas, with consistently high enrichment of excitatory neurons for B_P ratio images. Brainstem-normalized Schaefer 100 ^{13}C-metabolite GSEA was conducted in the same way as it was for the Schaefer 200 atlas, and both model-based and spin permutation-based results can be found in Tables S7 and S8, respectively,

although there are no significant enrichments.

Table S5: GSEA results for Schaefer 100 ^{13}C-ratios using model-based null distributions.

	B_P		L_P		L_B	
**Cell Type**	**ES**	***p***	**ES**	***p***	**ES**	***p***
Ast	−0.26	0.60	0.34	0.32	0.43	0.13
End	0.39	0.086	−0.16	0.89	−0.29	0.25
Ex1	0.41	0.17	0.22	0.81	−0.22	0.81
Ex2	0.46	0.036	0.25	0.56	−0.21	0.79
Ex3a	0.41	0.37	−0.33	0.64	−0.44	0.33
Ex3b	0.46	0.095	−0.24	0.75	−0.38	0.21
Ex3c	0.47	0.064	0.20	0.81	−0.27	0.52
Ex3d	0.33	0.53	−0.39	0.37	−0.48	0.095
Ex3e	0.50	0.087	0.37	0.37	0.28	0.62
Ex4	0.47	0.095	−0.26	0.73	−0.40	0.25
Ex5a	0.48	0.029	−0.18	0.92	−0.29	0.41
Ex5b	0.44	0.046	0.27	0.42	0.21	0.78
Ex6a	0.42	0.032	0.27	0.34	−0.21	0.70
Ex6b	0.43	0.062	0.31	0.22	0.28	0.35
Ex8	0.46	0.056	0.37	0.15	0.27	0.46
In1a	0.27	0.38	0.31	0.15	0.29	0.23
In1b	0.28	0.28	0.30	0.16	0.25	0.39
In1c	0.31	0.18	0.29	0.22	0.23	0.58
In2	0.25	0.46	0.20	0.82	−0.20	0.85
In3	0.24	0.64	−0.22	0.79	−0.29	0.47
In4a	0.27	0.41	0.33	0.14	0.30	0.23
In4b	0.29	0.16	0.24	0.31	0.21	0.55
In6a	0.23	0.60	−0.29	0.29	−0.33	0.14
In6b	0.21	0.81	−0.37	0.28	−0.40	0.21
In7	0.20	0.82	−0.23	0.70	−0.21	0.84
In8	0.32	0.14	0.29	0.16	0.28	0.20
Mic	0.27	0.26	0.34	0.096	0.25	0.35
OPC	0.19	0.80	0.33	0.22	0.37	0.12
Oli	0.46	0.14	0.38	0.29	0.16	0.88
Per	0.37	0.12	0.36	0.14	0.26	0.38

Table S6: GSEA results for Schaefer 100 ^{13}C-ratios using spin permutation-based null distributions.

	B_P		L_P		L_B	
**Cell Type**	**ES**	***p***	**ES**	***p***	**ES**	***p***
Ast	0.16	0.58	0.34	0.44	0.41	0.22
End	0.46	**0.0099**	0.16	0.58	−0.30	0.057
Ex1	0.40	**0.16**	0.23	0.26	−0.20	0.68
Ex2	0.45	**0.096**	0.26	0.20	−0.20	0.50
Ex3a	0.36	**0.10**	−0.32	0.84	−0.43	0.56
Ex3b	0.45	**0.16**	−0.23	0.58	−0.37	0.48
Ex3c	0.48	**0.15**	0.20	0.32	−0.27	0.44
Ex3d	0.29	**0.19**	−0.39	0.92	−0.49	0.49
Ex3e	0.52	0.37	0.37	0.86	0.28	0.76
Ex4	0.45	**0.15**	−0.26	0.54	−0.40	0.58
Ex5a	0.47	0.26	0.19	0.50	−0.29	0.59
Ex5b	0.42	**0.12**	0.28	0.38	0.23	0.71
Ex6a	0.38	**0.11**	0.26	0.27	−0.21	0.63
Ex6b	0.40	**0.096**	0.31	0.42	0.28	0.46
Ex8	0.44	**0.13**	0.37	0.75	0.28	0.58
In1a	0.31	**0.19**	0.32	0.54	0.30	0.58
In1b	0.29	**0.16**	0.30	0.44	0.25	0.50
In1c	0.32	**0.13**	0.28	0.25	0.23	0.58
In2	0.26	**0.16**	0.22	0.29	−0.20	0.53
In3	0.24	**0.21**	−0.23	0.23	−0.29	0.38
In4a	0.29	**0.16**	0.34	0.57	0.31	0.64
In4b	0.31	**0.21**	0.24	0.26	0.22	0.65
In6a	0.24	**0.22**	−0.30	0.55	−0.33	0.52
In6b	0.23	**0.14**	−0.37	0.64	−0.41	0.40
In7	0.22	**0.16**	−0.23	0.35	−0.21	0.51
In8	0.34	**0.14**	0.29	0.41	0.27	0.40
Mic	0.32	**0.046**	0.33	0.057	0.23	0.10
OPC	0.24	**0.14**	0.34	0.31	0.36	0.41
Oli	0.53	**0.16**	0.36	0.52	0.16	0.60
Per	0.49	**0.0069**	0.35	0.092	0.22	0.091

Table S7: GSEA results for Schaefer 100 brainstem-normalized ^{13}C-metabolites using model-based null distributions.

	^{13}C-BICARBONATE		^{13}C-LACTATE		^{13}C-PYRUVATE	
**Cell Type**	**ES**	***p***	**ES**	***p***	**ES**	***p***
Ast	0.43	0.11	−0.42	0.12	−0.34	0.42
End	−0.29	0.28	0.21	0.62	0.17	0.80
Ex1	−0.22	0.81	0.27	0.63	−0.16	0.97
Ex2	−0.21	0.79	0.28	0.29	−0.19	0.89
Ex3a	−0.44	0.26	0.48	0.18	0.34	0.69
Ex3b	−0.38	0.21	0.32	0.37	0.17	0.96
Ex3c	−0.27	0.56	0.17	0.90	−0.24	0.58
Ex3d	−0.48	0.083	0.49	0.078	0.40	0.32
Ex3e	0.28	0.69	−0.30	0.63	−0.40	0.27
Ex4	−0.40	0.21	0.40	0.22	0.24	0.82
Ex5a	−0.29	0.40	0.26	0.48	−0.15	0.98
Ex5b	0.21	0.75	0.22	0.69	−0.21	0.80
Ex6a	−0.21	0.68	0.26	0.30	0.17	0.94
Ex6b	0.28	0.35	0.22	0.62	−0.17	0.95
Ex8	0.27	0.47	−0.17	0.95	−0.28	0.36
In1a	0.29	0.24	−0.17	0.91	−0.21	0.69
In1b	0.25	0.40	0.23	0.57	0.18	0.90
In1c	0.23	0.62	0.27	0.36	0.22	0.62
In2	−0.20	0.85	0.31	0.18	0.27	0.31
In3	−0.29	0.44	0.36	0.17	0.32	0.28
In4a	0.30	0.21	0.15	0.97	−0.23	0.57
In4b	0.21	0.55	0.21	0.51	0.18	0.85
In6a	−0.33	0.15	0.33	0.12	0.29	0.27
In6b	−0.40	0.23	0.40	0.18	0.38	0.22
In7	−0.21	0.84	0.32	0.29	0.29	0.39
In8	0.28	0.22	0.21	0.60	−0.19	0.86
Mic	0.25	0.36	−0.23	0.43	−0.25	0.29
OPC	0.37	0.10	−0.31	0.28	−0.29	0.36
Oli	0.16	0.89	−0.11	0.99	−0.27	0.53
Per	0.26	0.46	−0.28	0.39	−0.25	0.45

Table S8: GSEA results for Schaefer 100 brainstem-normalized ^{13}C-metabolites using spin permutation-based null distributions.

	^{13}C-bicarbonate		^{13}C-lactate		^{13}C-pyruvate	
**Cell Type**	**ES**	***p***	**ES**	***p***	**ES**	***p***
Ast	−0.42	0.25	−0.39	0.45	−0.34	0.56
End	0.21	0.24	0.16	0.58	0.17	0.43
Ex1	0.27	0.67	0.24	0.80	−0.16	0.91
Ex2	0.28	0.52	0.23	0.84	−0.19	0.81
Ex3a	0.48	0.28	0.35	0.80	0.34	0.87
Ex3b	0.32	0.77	0.14	0.99	0.17	0.99
Ex3c	0.17	0.86	−0.26	0.76	−0.24	0.85
Ex3d	0.49	0.42	0.36	0.97	0.40	0.90
Ex3e	−0.30	0.65	−0.35	0.94	−0.40	0.71
Ex4	0.40	0.58	0.24	0.85	0.24	0.90
Ex5a	0.26	0.65	−0.13	0.99	−0.15	0.87
Ex5b	0.22	0.76	0.19	0.88	−0.21	0.88
Ex6a	0.26	0.53	0.22	0.86	0.17	0.89
Ex6b	0.22	0.55	0.27	0.84	−0.17	0.81
Ex8	−0.17	0.90	0.20	0.89	−0.28	0.80
In1a	−0.17	0.85	0.14	0.98	−0.21	0.92
In1b	0.23	0.73	0.26	0.85	0.18	0.92
In1c	0.27	0.49	0.30	0.82	0.22	0.92
In2	0.31	0.46	0.28	0.83	0.27	0.86
In3	0.36	0.42	0.35	0.61	0.32	0.87
In4a	0.15	0.85	0.20	0.83	−0.23	0.86
In4b	0.21	0.74	0.20	0.91	0.18	0.93
In6a	0.33	0.52	0.26	0.81	0.29	0.90
In6b	0.40	0.46	0.29	0.81	0.38	0.72
In7	0.32	0.53	0.35	0.88	0.29	0.86
In8	0.21	0.59	0.25	0.83	−0.19	0.73
Mic	−0.23	0.31	−0.22	0.35	−0.25	0.27
OPC	−0.31	0.36	−0.21	0.58	−0.29	0.57
Oli	−0.11	0.96	−0.14	0.94	−0.27	0.69
Per	−0.28	0.21	−0.27	0.21	−0.25	0.21
